# Exploiting the noise: improving biomarkers with ensembles of data analysis methodologies

**DOI:** 10.1186/gm385

**Published:** 2012-11-12

**Authors:** Maud HW Starmans, Melania Pintilie, Thomas John, Sandy D Der, Frances A Shepherd, Igor Jurisica, Philippe Lambin, Ming-Sound Tsao, Paul C Boutros

**Affiliations:** 1 Informatics and Biocomputing Platform, Ontario Institute for Cancer Research, Toronto, ON, M5G 0A3, Canada; 2 Department of Radiation Oncology (Maastro), GROW-School for Oncology and Developmental Biology, Maastricht University Medical Center, Maastricht, The Netherlands; 3 Ontario Cancer Institute and the Campbell Family Institute for Cancer Research, University Health Network, Toronto, ON, M5G 2M9, Canada; 4 Ludwig Institute for Cancer Research, Austin Health, Melbourne, Australia; 5 Department of Medical Oncology and Hematology, Princess Margaret Hospital, University Health Network, Toronto, ON, M5G 2M9, Canada; 6 Department of Medical Biophysics, University of Toronto, Toronto, ON, M5G 2M9, Canada; 7 Techna Institute, University Health Network, Toronto, ON, M5G 2M9, Canada; 8 Department of Computer Science, University of Toronto, Toronto, ON, M5G 2M9, Canada

## Abstract

**Background:**

The advent of personalized medicine requires robust, reproducible biomarkers that indicate which treatment will maximize therapeutic benefit while minimizing side effects and costs. Numerous molecular signatures have been developed over the past decade to fill this need, but their validation and up-take into clinical settings has been poor. Here, we investigate the technical reasons underlying reported failures in biomarker validation for non-small cell lung cancer (NSCLC).

**Methods:**

We evaluated two published prognostic multi-gene biomarkers for NSCLC in an independent 442-patient dataset. We then systematically assessed how technical factors influenced validation success.

**Results:**

Both biomarkers validated successfully (biomarker #1: hazard ratio (HR) 1.63, 95% confidence interval (CI) 1.21 to 2.19, *P *= 0.001; biomarker #2: HR 1.42, 95% CI 1.03 to 1.96, *P *= 0.030). Further, despite being underpowered for stage-specific analyses, both biomarkers successfully stratified stage II patients and biomarker #1 also stratified stage IB patients. We then systematically evaluated reasons for reported validation failures and find they can be directly attributed to technical challenges in data analysis. By examining 24 separate pre-processing techniques we show that minor alterations in pre-processing can change a successful prognostic biomarker (HR 1.85, 95% CI 1.37 to 2.50, *P *< 0.001) into one indistinguishable from random chance (HR 1.15, 95% CI 0.86 to 1.54, *P *= 0.348). Finally, we develop a new method, based on ensembles of analysis methodologies, to exploit this technical variability to improve biomarker robustness and to provide an independent confidence metric.

**Conclusions:**

Biomarkers comprise a fundamental component of personalized medicine. We first validated two NSCLC prognostic biomarkers in an independent patient cohort. Power analyses demonstrate that even this large, 442-patient cohort is under-powered for stage-specific analyses. We then use these results to discover an unexpected sensitivity of validation to subtle data analysis decisions. Finally, we develop a novel algorithmic approach to exploit this sensitivity to improve biomarker robustness.

## Background

The perfect medical treatment would start with instantaneous diagnosis, proceed rapidly to a treatment that offered complete cure with no side effects, and of course would incur minimal costs to the healthcare system. This dream scenario, while distant, has been made more plausible by a rapid reduction in the cost of molecular assays. Molecular profiling of clinical specimens offers hope in two ways. First, new candidate therapeutic targets are being identified [1,2]. These may lead to new treatments that cure diseases more reliably and rapidly, and have fewer side effects than existing approaches. Second, molecular profiling is leading to the development of biomarkers that can identify the optimal therapy for an individual patient [3]. Together, these two trends are enabling molecularly personalized medicine: biomarkers are used to select optimal treatments from a large repertoire.

Unfortunately, the field of biomarker development has not reached its translational potential. Despite numerous reports of molecularly derived biomarkers to diagnose disease [4], predict prognosis for individual patients [5], and forecast response to therapy [6], the majority of biomarkers do not reach clinical use. The reasons for this are numerous. Some groups have made major statistical errors in deriving their biomarkers [7,8]. Others have failed to adjust for key clinical information, such as stage, or have failed to demonstrate their approaches are superior to existing, non-molecular methodologies [9-11]. Sometimes, external validation studies are entirely missing. But hundreds of biomarkers have been developed avoiding these concerns, but still fail in external validation studies [12]. This has long been thought to reflect the high dimensionality and complexity of biomarker space [13,14].

The management of resectable non-small cell lung cancer (NSCLC) would particularly benefit from the development of new prognostic tools. Despite improvements in staging, surgical methodologies, chemotherapy regimens and the addition of adjuvant therapies, 30 to 50% of patients with resectable NSCLC suffer relapse and die within 5 years [15-17]. In fact, assessments of tumor size and spread (TNM staging) are still the predominant prognostic variables in use. Several groups, including our own, have employed transcriptome profiling of surgically excised tumor samples to develop multi-gene prognostic biomarkers (sometimes called prognostic signatures or classifiers) [5,6,9,10,18,19]. However, there is minimal gene-wise overlap between these multi-gene biomarkers [20], and challenges exist in the datasets and analyses used to generate them [12].

Given the clinical need for a robust prognostic biomarker for NSCLC and the technical challenges that confounded prior studies, a multi-institute effort was undertaken. The Director's Challenge NSCLC study was an attempt to provide a large, sufficiently powered dataset to discover reproducible multi-gene biomarkers [21]. This consortium integrated four independent datasets of adenocarcinomas named according to the institutions at which they were generated: UM, HLM, MSK and CAN/DF. Each analysis group was blinded to the validation cohort, and developed independent biomarkers that were compared to the appropriate clinical end-points. In 2008 the Director's Challenge team reported the surprising finding that none of the multi-gene biomarkers tested were validated for the primary end-point of stage I survival, supporting the idea that large validation cohorts are required. More recently, Subramanian and Simon [12] performed a critical review of a number of prognostic multi-gene biomarkers for NSCLC. They attempted to validate several previously published biomarkers on the Director's Challenge dataset, and again found that no prognostic biomarker validated in this large (442-patient) independent cohort.

This study begins with an attempt to replicate the results of Subramanian and Simon [12]. Surprisingly, we are unable to do so: by following the exact procedures used in the original studies we show that both prognostic biomarkers tested actually validate in the Director's Challenge cohort [21]. This is an unexpected positive finding that led us to systematically evaluate the reasons for this discrepancy. We first show that even this large cohort is underpowered for stage-specific analyses. We then find that relatively subtle changes in data-analysis greatly confound biomarker validation. By exploring this phenomenon we show that this is a general feature of multiple datasets and biomarkers. Finally, we show that the 'noise' caused by changes in analysis protocols is actually an important source of information about the robustness of a biomarker and provide a novel algorithm that exploits it.

## Materials and methods

### Classifier evaluation

All analyses were performed in the R statistical environment (v2.11.1). The Director's Challenge datasets [21] were used to validate two previously published biomarkers, one containing three genes [20] and the other containing six genes [13]. Data pre-processing and patient classification were performed exactly as in the original studies [13,20]. The Director's Challenge consists of four independent datasets; UM, HLM, MSK and CAN/DF. Because Shedden *et al. *[21] reported high inter-group variability, these datasets were pre-processed separately with the RMA algorithm [22] (R package: affy v1.28.0).

On the microarrays used for the Director's Challenge study every gene is represented by a set of 25 bp oligonucleotides, called a ProbeSet. ProbeSet annotation was done with Affymetrix provided annotation (R packages: hgu133aprobe v2.6.0 and hgu133acdf v2.6.0). The exact ProbeSets used in the original study were evaluated (Table S1 in Additional file 1). Median scaling and housekeeping gene normalization (to the geometric mean of *ACTB, BAT1, B2M *and *TBP *levels) on biomarker genes was performed before statistical modeling to generate normalized expression values, as for the original classifiers [13,20].

The three-gene classifier contains the genes *CCR7, HIF1A *and *STX1A*. The normalized expression values for these genes were subjected to statistical scaling and then median-dichotomized, as outlined in Lau *et al. *[20]. A risk score was then calculated from the scaled, normalized expression as:

$$\text{RiskScore}=4\times \text{STX}1{\text{A}}_{\text{expr}}+3\times \text{HIF}1{\text{A}}_{\text{expr}}-3\times \text{CCR}7_{\text{expr}}$$

In this equation STX1A_expr _for a patient is set to one if their STX1A signal intensity (after all pre-processing) is above the median for all patients in the dataset and zero otherwise. Values for HIF1A_expr _and CCR7_expr _are calculated analogously. Patients were classified into risk groups based on their risk score: patients with a score ≤ 2 were predicted to have good prognosis, whereas those with scores above 2 were predicted to have poor prognosis, as in the original report of this biomarker [20].

For the six-gene classifier, Euclidean distances to the training cluster centers computed and reported in the original study were calculated to classify each patient [13]. Briefly, the distance between a patient's profile and the cluster center was calculated separately for each cluster. The ratio of these two distances was then assessed: if it was between 0.9 and 1.11, the patient-classification was deemed ambiguous. These patients were left unclassified and were ignored in downstream analyses. All other patients were classified into the nearer of the two clusters. These procedures are identical to those originally reported for this classifier [13].

Prognostic performance of both classifiers was evaluated in three ways: Kaplan-Meier survival curves, stage-adjusted Cox proportional hazard ratio modeling followed by the Wald test (R package: survival v2.35-8) and binary classification measures. Overall survival was used as the primary endpoint; therefore, survival was truncated at five-years for these analyses, since death due to other causes increases considerably after 5 years in lung cancer survivors: if an event occurred after 5 years, it was ignored and the survival time was set to 5 years. For binary classification performance, patients assigned to the poor prognosis group were considered true positives (TP) if they died within 5 years, whereas if these patients survived longer than 5 years they were called false positives (FP). Likewise, patients alive at 5 years and classified in the good prognosis group were considered true negatives (TN); however, if they died within 5 years they were considered false negatives (FN). Patients with data censoring before 5 years were disregarded for these analyses (84 patients). These numbers were used to calculate sensitivity, specificity and accuracy in the usual ways.

### Power analysis

A power analysis [23] was performed to estimate the likelihood that differences could be identified in stage-dependent patient subgroups (R package: Hmisc v3.8-2). For power calculations we assume equal numbers of patients in each risk group, as previously observed for the three-gene marker [20]. Power was calculated as a function of the number of events (NE), in this case deaths, hazard ratio (HR) and the significance level (α) as derived by Schoenfeld [23]:

$$z_{power}=\frac{\sqrt{NE}\times \text{log}\left(HR\right)}{2}-z_{1-\frac{\alpha }{2}}$$

The probability of finding a specific HR given a certain NE and using a significance level of α is calculated from Z_power_. For our analysis we set α = 0.05 and evaluated the Director's Challenge cohort as a whole (442 patients; 236 events) or by only considering stage IA (114 patients; 38 events), stage IB (162 patients; 73 events), stage II (95 patients; 64 events), or stage III patients alone (68 patients; 60 events). We calculated the power for each cohort at a range of HRs from 1 to 2.5 in 150 increments of 0.01.

### Dataset pre-processing sensitivity assessment

To assess the influence of different pre-processing schedules on signature performance, the Director's Challenge datasets were pre-processed in 24 different ways and both classifiers were evaluated in each of these datasets. We considered four separate factors. Here we outline each of the pre-processing options evaluated.

First pre-processing for the four datasets was either done for all datasets separately or for all combined. When datasets were treated separately, pre-processing was performed independently for each of the four datasets. Patient groups were then predicted separately for each cohort: patients predicted as having good prognosis (independent of the dataset they originated from) were pooled into one group and those predicted as having poor prognosis (again, independent of dataset) were pooled into another group. Alternatively, when all datasets were combined the raw data were merged for a single pre-processing.

Second, four different, commonly used pre-processing algorithms were applied: RMA [22], GCRMA [24], MAS5 [25,26] and MBEI [27] (R packages: affy v1.26.1, gcrma v2.20.0). Table S2 in Additional file 1 gives a brief description of each of these algorithms.

Third, while RMA and GCRMA provide data in log_2_-transformed space, MAS5 and MBEI provide data in normal space. It is common, but not universal, to log_2_-transform MAS5 and MBEI pre-processed data. We therefore tested these two algorithms in both normal and log_2_-space.

Fourth, the annotation of individual ProbeSets has changed significantly as our understanding of the human transcriptome has evolved. Modern microarray analyses typically address this by using updated ProbeSet annotations, such as those created by Dai *et al. *[28]. We evaluated the effect of using these alternatives to standard annotation procedures. ProbeSet annotation was done with both 'default' (R packages: hgu133aprobe v2.6.0 and hgu133acdf v2.6.0) and updated Entrez Gene-based 'alternative' annotations [28] (R packages: hgu133ahsentrezgprobe v12.1.0 and hgu133ahsentrezgcdf v12.1.0).

We re-processed the data using all possible methods. This meant that we conducted 24 separate analyses, once for each possible combination of 6 (4 without log_2_-transformation + 2 with log_2_-transformation) pre-processing algorithms, 2 ProbeSet annotation techniques and 2 dataset handling approaches. When the default Affymetrix gene-annotation was applied, the corresponding Affymetrix ProbeSets from the original study were used. When the alternative Entrez Gene ID ProbeSet annotation was utilized, matching was performed based on Entrez Gene ID. Table S1 in Additional file 1 lists the specific ProbeSets used for each gene according to each annotation protocol. Additional file 2 and Additional file 3 give the key clinical data for each patient, along with the good/poor classifications for the three-gene and six-gene classifier in each of the pre-processing methods. These data allow complete recapitulation of all analyses presented here.

To test the generality of our findings, this procedure was applied identically to the Bild dataset [29] (R packages: affy v1.28.0, gcrma v2.22.0). This dataset consists of 2 batches; therefore the same 24 pre-processing schedules were applied. Default and alternative ProbeSet annotation were performed with the appropriate R packages (default: hgu133plus2probe v2.7.0, hgu133plus2cdf v2.7.0; alternative: hgu133plus2hsentrezgprobe v13.0.0, hgu133plus2hsentrezgcdf v13.0.0). The specific ProbeSets used for each gene according to each annotation protocol are listed in Table S1 in Additional file 1. Additional file 4 gives the key clinical data for each patient, along with the good/poor classifications for the three-gene classifier in each of the pre-processing methods.

Finally, to determine whether our observations were a function of the classification algorithm, we performed univariate analysis relating the signal intensity of each ProbeSet in the Director's Challenge dataset to patient outcome. Each individual ProbeSet was used to median-dichotomize the patient cohort and prognostic performance was evaluated with an unadjusted Cox proportional hazard ratio modeling followed by the Wald test. This was repeated again for the 24 different procedures noted above.

### Visualizations

All plotting was performed in the R statistical environment (v2.15.1) using the lattice (v0.20-6), latticeExtra (v0.6-19), RColorBrewer (v1.0-5) and cluster (v.1.14.2) packages.

## Results

### Validation of two NSCLC prognostic biomarkers

We first sought to replicate and extend the results of Subramanian and Simon [12], who reported that two prognostic biomarkers for NSCLC, including a three-gene one [20], did not validate in the 442-patient Director's Challenge cohort [21]. Following the exact procedures described in the original studies, we attempted to validate both this three-gene biomarker and another six-gene [13] prognostic biomarker in the Director's Challenge cohort. These two biomarkers were derived using distinct methodologies: one using a linear risk-score analysis and the other using gradient-descent-based optimization. Neither used the Director's Challenge datasets for training.

**Figure 1:**
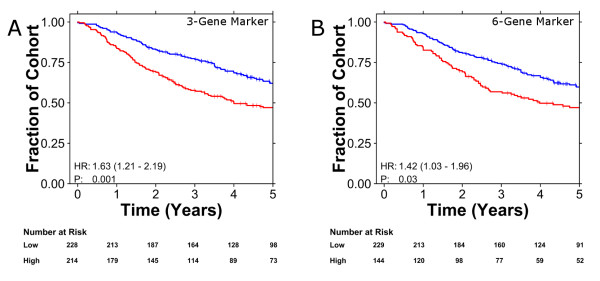
**Validation of three- and six-gene biomarkers**. Previously published three-gene [20] and six-gene [13] prognostic biomarkers were validated in the Director's Challenge dataset [21]. **(a, b) **Each patient was classified into good (blue curves) or poor (red curves) prognosis groups using the three-gene (a) and six-gene (b) biomarker, which were visualized with Kaplan-Meier curves. Hazard ratios and *P*-values are from stage-adjusted Cox proportional hazard ratio modeling followed by the Wald test.

We assessed performance using stage-adjusted Cox proportional hazards models. Both the three-gene biomarker (Figure 1a; HR 1.63, 95% CI 1.21 to 2.19, *P *= 1.18 × 10^-3^, Wald test; 54% accuracy) and the six-gene biomarker (Figure 1b; HR 1.42, 95% CI 1.03 to 1.96, *P *= 3.01 × 10^-2^, Wald test; 46% accuracy) identified poor-prognosis patients in a stage-independent manner, albeit with modest effect sizes. This is an exciting result: it reflects the second and third large, independent validations of any NSCLC prognostic biomarkers [5] and, to the authors' knowledge, the first two biomarkers to classify the Director's Challenge cohort into high- and low-risk groups that supplement pathological stage.

**Figure 2:**
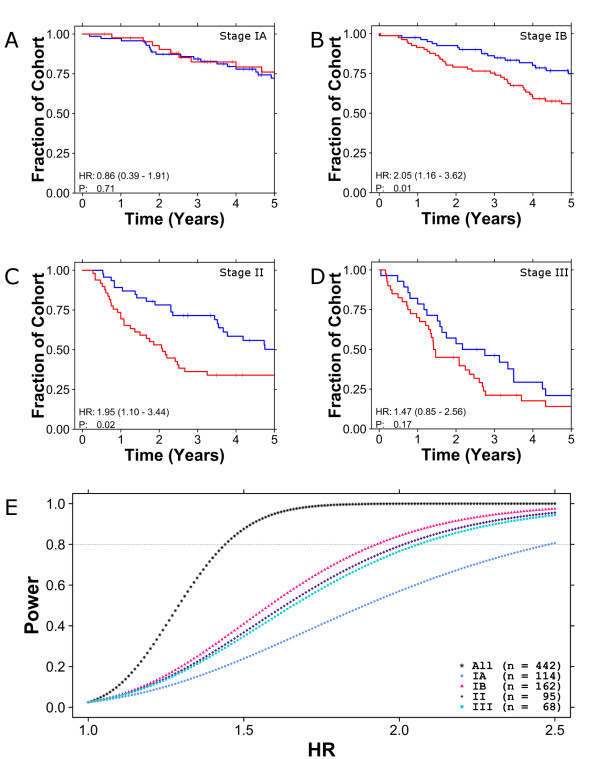
**Sub-stage analysis for three-gene biomarker and power analysis**. **(a-d) **The performance of the three-gene biomarker was evaluated in a sub-stage analysis (stage IA (a), stage IB (b), stage II (c) and stage III (d) patients). Each patient was classified into good (blue curves) and poor (red curves) prognosis groups using the three-gene biomarker and were displayed with Kaplan-Meier curves. Hazard ratios and *P*-values are from Cox proportional hazard ratio modeling followed by the Wald test. **(e) **Subsequently, power calculations (assuming equal-sized groups) were performed at a range of HRs for all patients and for patients of specific stages. A threshold line is drawn for power of 0.8.

We then proceeded, as did Subramanian and Simon, by performing sub-group analysis on individual stages, with a focus on stage IB patients (who might derive benefit from additional treatment) and stage II patients (who might be over-treated). Both biomarkers were ineffective at classifying stage IA patients (three-gene biomarker (Figure 2a): HR 0.86, *P *= 0.710, 52% accuracy; six-gene biomarker (Additional file 5): HR 0.69, *P *= 0.42, 50% accuracy). The three-gene biomarker did validate in stage IB (Figure 2b; HR 2.05, *P *= 1.41 × 10^-2^, 58% accuracy) and stage II patients (Figure 2c; HR 1.95, *P *= 2.11 × 10^-2^, 60% accuracy), although not in stage III patients (Figure 2d). The six-gene biomarker showed a trend for stage IB patients, successfully stratified stage II patients, and failed for stage III patients (Additional file 5b-d; Table S3 in Additional file 1). These results suggest that each of the biomarkers shows promise in the clinically relevant sub-groups, but with stage-specific trends. Notably, effect sizes are largest for the clinically critical stage IB and II patients.

### A large, mixed-stage cohort is under-powered for sub-stage analysis

Next, to determine if the strong stage-specific trends observed are biologically meaningful, we performed a power analysis to estimate the likelihood that real differences could be identified in each group. Figure 2e shows the power (y-axis) as a function of the HR for each stage of the Director's Challenge cohort. The overall 442-patient cohort is robustly powered to identify biomarkers with a HR of 1.5, or higher. By contrast, most of the sub-stage analyses are marginally powered. For example, there is only a 57% chance of detecting a real hazard ratio of 2.0 in stage IA patients. This immediately rationalizes the stage-dependence of biomarker validation (Figures 2a-d): both biomarkers failed to stratify the two least-powered stages (IA and III - there are only 68 stage III patients), but were successful in the two better-powered stages (IB and II) and in the overall cohort. Importantly, stages IB and II are the most clinically relevant group for prognostic biomarkers for NSCLC.

### Prognostic biomarkers are highly sensitive to data pre-processing

The results outlined above do not validate those reported by Subramanian and Simon [12], who reported validation failures for the three-gene marker both in the overall cohort and in sub-stage analyses. We sought to rationalize the discrepancies between the two studies. After careful analysis we identified slight differences between the data pre-processing used by Subramanian and Simon and that described in the original studies (and used here). We pre-processed the data using a standard approach called robust multi-array (RMA). Each site-specific cohort was processed independently and patient-level results were merged for survival analysis. By contrast, Subramanian and Simon [12] used an alternative strategy called model-based expression indices (MBEI), with pseudo-count addition and merging of the four datasets prior to pre-processing, along with other minor changes. We replicated the alternative approach and found that the critical change was the change in pre-processing strategy: neither the three-gene biomarker (Additional file 6a; HR 1.28, *P *= 9.70 × 10^-2^; 54% accuracy) nor the six-gene biomarker (Additional file 6b; HR 1.00, *P *= 0.985; 38% accuracy) validated in the overall cohort. Similarly, they failed in the critical sub-stage analyses (Additional file 6c).

We were surprised that such a small deviation would affect biomarker validation so dramatically. To better understand the effect of different analysis strategies, we analyzed the Director's Challenge dataset using a panel of methods and evaluated both biomarkers against each. We investigated four separate factors. First, we compared treating the cohort as a single study or as four site-specific datasets (the original report indicated high inter-site variability [21]). Second, we employed four diverse and commonly-used pre-processing algorithms [22,24-27]. Third, we evaluated the effects of log_2_-transformation, a standard operation in microarray analysis. Finally, both default Affymetrix gene annotations and updated Entrez Gene-based annotations were tested [28]. We created 24 datasets by comparing all combinations of 2 dataset handling strategies, 6 (4 + 2) pre-processing algorithms and 2 annotation methods. We tested both prognostic biomarkers on each dataset for overall and stage-specific performance. Additional file 7 outlines this procedure; Additional files 2 and 3 give the classification of every patient using each of the 24 approaches.

**Figure 3:**
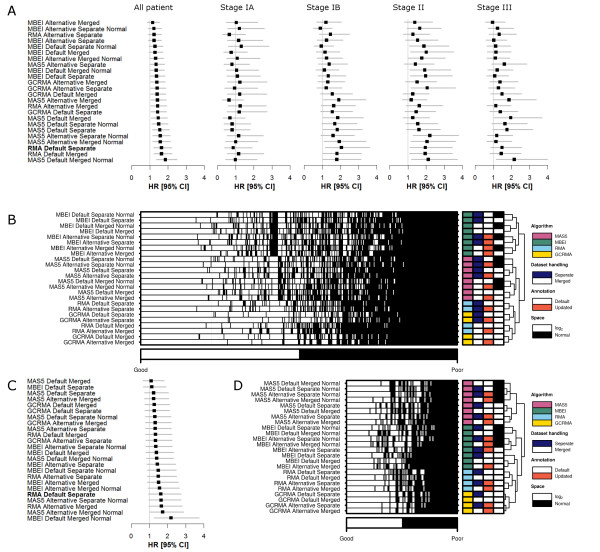
**Pre-processing influences biomarker validation**. **(a) **Results for all Cox proportional hazard ratio modeling analysis for the 24 different pre-processing schemes in the Director's Challenge dataset [21] are summarized in Forest plots. **(b) **Classifications in the 24 different schedules are visualized in a heatmap. To confirm that biomarker performance and individual patient classification are highly dependent on dataset pre-processing, all pre-processing schedules were tested in a second dataset [29] for the three-gene biomarker. **(c, d) **Both biomarker performance (c) and individual patient classifications (d) were again influenced by differences in pre-processing. For the Forest plots; boxes and lines are the hazard ratios and 95% confidence intervals, respectively. For the heatmaps; white indicates a patient predicted to have good prognosis and black indicates a patient predicted to have poor prognosis. Colored sidebar displays the different pre-processing schemes as explained in the legends.

This systematic analysis revealed that the validation of multi-gene biomarkers is highly sensitive to data pre-processing. This is especially true in stage-specific analyses: HRs for stage IB patients range from 0.89 (95% CI 0.52 to 1.53, *P *= 0.672) to 2.05 (95% CI 1.16 to 3.62, *P *= 1.41 × 10^-2^) for the three-gene classifier. Even in the overall cohort, small changes in pre-processing led to major changes in classification performance: sensitivity changed up to 14% and specificity 19% between methods (Tables S4 and S5 in Additional file 1). Within a single method, validation varied by stage: Figure 3a shows the approaches ranked by their performance in the overall cohort (three-gene biomarker), giving the HRs and their confidence intervals: sub-stage survival analyses are only weakly correlated to overall analysis. Importantly, no algorithm leads to validation in the under-powered stage IA group. This result was replicated with the six-gene biomarker (Table S6 and S7 in Additional file 1; Additional file 8a).

These differences in classifier performance are caused by changes in the classification status of a significant portion of patients. Figure 3b shows the classification status of each patient according to the three-gene biomarker (columns) for each schedule (rows). Patients annotated in black are classified as poor prognosis, and many cases are evident where different algorithms lead to different classifications. Only 151 out of 442 patients are classified identically by all 24 pre-processed schemes; these are equally in the good (77) and poor (74) prognosis groups. Again, the six-gene biomarker showed an identical trend (Additional file 8b).

To generalize this trend and to demonstrate that it is not an artifact of the Director's Challenge cohort, we repeated our analyses in an independent dataset [29]. The same variability across analysis methods was observed (Figure 3c). Only 45 out of 111 patients are classified identically across the 24 pre-processing methodologies using the three-gene biomarker (Figure 3d; Additional file 4), and there were large differences in validation rates (Tables S8 and S9 in Additional file 1).

### Univariate analyses are also susceptible to pre-processing effects

To determine whether this pre-processing sensitivity is generalizable, we performed univariate analyses for all individual ProbeSets in the Director's Challenge datasets. This analysis was repeated for each of the 24 pre-processing strategies (Additional file 7) [28]. The results are consistent: only 3.5% of genes as defined using the alternative annotation were significant in all pre-processing schemes (*P*-value Wald test ≤ 0.05). By contrast, approximately 40% of the genes were significantly associated with outcome in at least one pre-processing schedule, independent of the gene annotation used (Additional file 9).

### Pre-processing variability improves patient classifications

These data suggest that the use of publicly available patient cohorts for validation of both single- and multi-gene biomarkers, a very common practice, is fraught with challenges. The extreme sensitivity to data pre-processing means that minor errors can lead to entirely incorrect results. However, we wondered if statistical techniques could be developed to take advantage of the signals causing this variability. We reasoned that each analysis methodology might have a distinct error-profile and thus deviations reflect cases where small differences can change the assignment to a specific clinical group. As a result, they provide a measure of the robustness or informativeness of a molecular classification.

**Figure 4:**
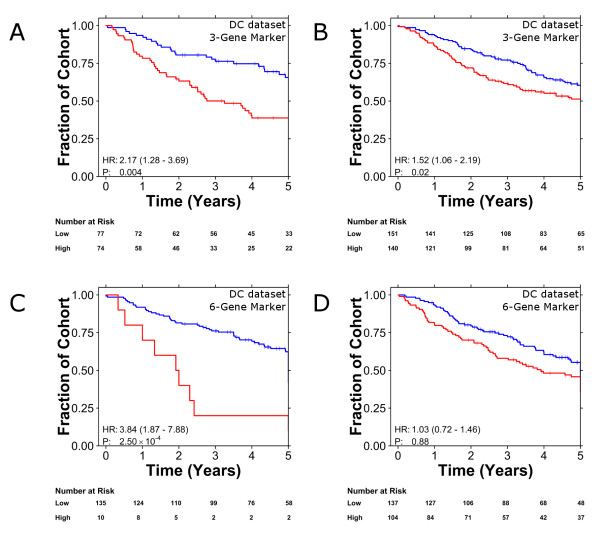
**Improved biomarker performance by accounting for classification robustness**. **(a-d) **Marker performance improved dramatically when differentiating patients with identical classifications across all pre-processing schemes from the patients with ambiguous classifications for both the three-gene biomarker (a versus b) and the six-gene biomarker (c versus d) in the Director's Challenge dataset [21]. For all Kaplan-Meier curves; good prognosis patients are indicated by blue curves and poor prognosis patients by red curves. Hazard ratios and *P*-values are from stage-adjusted Cox proportional hazard ratio modeling followed by the Wald test.

To exploit this source of information we treated the set of 24 pre-processing methodologies as an ensemble classifier. Each patient was treated as a vector of 24 predictions (one from each methodology), and unanimous classifications were treated as robust predictions while discordant classifications were treated as unreliable. In the Director's Challenge cohort this approach improved the performance of both the three- and six-gene biomarkers. The 151 patients with high-confidence predictions by the three-gene biomarker separate into clinically distinct groups (Figure 4a; HR 2.17, *P *= 4.00 × 10^-3^; 63% accuracy), while those with more ambiguous classifications, taking risk groups as assigned with the pre-processing schedule used in the original study [20], show a modest effect (Figure 4b; HR 1.52, *P *= 2.43 × 10^-2^; 55% accuracy). This trend is exaggerated for the six-gene biomarker, where the 145 patients with high-confidence predictions show a strong separation between good- and poor-prognosis groups, while the remaining patients show no trend (Figure 4c versus Figure 4d, HR = 3.84 versus HR = 1.03; Table S10 in Additional file 1). To generalize this approach, we replicated it in an independent cohort using the three-gene biomarker (Additional file 10a versus Additional file 10b; Table S10 in Additional file 1).

## Discussion

The development of robust biomarkers is critical for the delivery of highly personalized genomic medicine. Validation studies of biomarkers are typically under-powered [30], and often publicly available data are used to reduce expense and to avoid the challenge of finding patient cohorts with suitable clinical characteristics and annotation. We show here that the strategy of using public datasets can be fraught with unexpected challenges: biomarkers are extremely sensitive to analysis protocols.

When using author-prescribed analysis protocols exactly, both biomarkers tested here were successfully validated. In fact, one of the two biomarkers could sub-classify both stage IB and stage II patients into groups with significantly different survival properties, despite lack of power in these cohorts. These results emphasize the importance of continued validation on new datasets, as even the largest existing cohorts are insufficiently powered. Additionally, these results comprise the first reports of successful validation on the Director's Challenge dataset.

The surprising discordance of our results with those of Subramanian and Simon [12] reflect differences in analysis methodologies. They observed no validation for stage IB (*P *= 0.35), leading them to suggest that current multi-gene biomarkers lack clinical utility. We investigated the origins of this discrepancy and identified differences in microarray data pre-processing. Instead of RMA-normalized data with each site in the Director's Challenge treated separately, Subramanian and Simon [12] used MBEI quantile-normalized data with pseudo-count addition, while treating all sites together. A similar issue has been noted in other studies where differences in pre-processing contributed to failed reproduction of drug sensitivity predictions [31,32]. Our findings are analogous to other reports that pre-processing influences other downstream analyses [22,33,34]. Observed discrepancies can be attributed to differences in the pre-processing algorithms, but will also likely depend on the dataset(s) and marker(s) that are evaluated. For example, background correction is handled completely differently using individual algorithms (Table S2 in Additional file 1). Indeed, the optimal pre-processing method is intrinsically dependent on experimental design and cannot be standardized [34,35].

Yet, we also show that this sensitivity actually carries important information. A useful biomarker ought not be highly sensitive to small perturbations. Real clinical samples are subject to variable and poorly controlled factors like the length of time prior to freezing or fixation post-surgery or the degree of stromal contamination. We show that the comparison of different analysis methodologies can be used as a measure of confidence in the predictions of a biomarker. This immediately provides a low-cost and rapid method of improving any existing biomarker, and will be a major boon for the more complex biomarkers emerging from next-generation sequencing studies.

We also show that these successful biomarkers only performed well for a fraction of patients. For example, the six-gene biomarker worked extraordinarily well for 145 of the 442 patients in the Director's Challenge cohort, but failed entirely on the remainder (Figure 4c, d). Similarly, the three-gene biomarker performed very well on 151 patients, but poorly on the others. Surprisingly, classification overlap occurred in only 68 patients between these two groups. This may suggest that a battery of multi-gene biomarkers will be required, with each performing well on some patients but not others. These data indicate that individualized medicine will require personalized biomarkers.

## Conclusions

We report the successful validation of two prognostic biomarkers for NSCLC in the 442-patient Director's Challenge dataset. Despite using an underpowered dataset, these biomarkers significantly prognosed clinically relevant patient sub-groups. In the course of this validation we discovered an extreme sensitivity to the pre-processing methodology. The importance of such an effect goes against dogma in the field: it was recently stated that 'The differences in the preprocessing steps for microarray data are immaterial... [when] the original classifier was developed using RT-qPCR' [36]. Our results demonstrate that this statement is incorrect. Instead, there is significant noise caused by pre-processing, but ensemble methods can be used to exploit this noise to improve our ability to personalize treatment decisions at no experimental cost.

## Abbreviations

HR: hazard ratio; CI: confidence interval; MBEI: model-based expression indices; NE: number of events; NSCLC: non-small cell lung cancer; RMA: robust multi-array averaging; GCRMA: GeneChip robust multi-array averaging; MAS5: microarray analysis suite 5.0.

## Competing interests

The authors declare that they have no competing interests.

## Authors' contributions

All authors were involved in the study design. Data were collected and analyzed by MHWS, MP and PCB. Statistical analysis was performed by MHWS, MP and PCB. Data interpretation was performed by MHWS, MP, MST and PCB. The manuscript was initially drafted by MHWS and PCB. All authors contributed to drafting of the manuscript. All authors have read and approved the manuscript for publication.

## Supplementary Material

Additional file 1 **Supplementary tables**. Table S1: ProbeSet annotation used in analyses. Supplementary Table S2: overview of pre-processing algorithms. Supplementary Table S3: results of binary prediction performance of the three-gene and six-gene classifier in the Director's Challenge dataset. Supplementary Table S4: results (stage-adjusted) Cox proportional hazard ratio modeling three-gene classifier for all patients and stage IB patients in the 24 different pre-processed Director's Challenge datasets. Significant results (*P *< 0.05) are given in bold. Supplementary Table S5: results of binary prediction performance of the three-gene classifier in the 24 different pre-processed Director's Challenge datasets. Supplementary Table S6: results (stage-adjusted) Cox proportional hazard ratio modeling six-gene classifier for all patients and stage II patients in the 24 different pre-processed Director's Challenge datasets. Significant results (*P *< 0.05) are given in bold. Supplementary Table S7: results of binary prediction performance of the six-gene classifier in the 24 different pre-processed Director's Challenge datasets. Supplementary Table S8: results (stage-adjusted) Cox proportional hazard ratio modeling three-gene classifier for all patients in the 24 different pre-processed Bild datasets. Significant results (*P *< 0.05) are given in bold. Supplementary Table S9: results of binary prediction performance of the three-gene classifier in the 24 different pre-processed Bild datasets. Supplementary Table S10: results of binary prediction performance of the two classifiers in the Director's Challenge and Bild datasets for unanimous and ambiguous classified patients. Click here for file

Additional file 2 **Clinical information and good/poor classification for the three-gene classifier in each of the pre-processing methods in the Director's Challenge datasets**. Click here for file

Additional file 3 **Clinical information and good/poor classification for the six-gene classifier in each of the pre-processing methods in the Director's Challenge datasets**. Click here for file

Additional file 4 **Clinical information and good/poor classification for the three-gene classifier in each of the pre-processing methods in the Bild datasets**. Click here for file

Additional file 5 **Supplementary Figure S1**. **(a-d) **Performance of the six-gene biomarker was evaluated in a sub-stage analysis (stage IA (a), stage IB (b), stage II (c), and stage III (d) patients), which were visualized with Kaplan-Meier curves. Each patient was classified into good (blue curves) and poor (red curves) prognosis groups using the six-gene biomarker. Hazard ratios and *P*-values are from Cox proportional hazard ratio modeling followed by the Wald test. Click here for file

Additional file 6 **Supplementary Figure S2**. **(a-d) **Kaplan-Meier curves for the three-gene (a: all patients; c: stage IB) and six-gene (b: all patients; d: stage II) classifiers in the Director's Challenge data [21], where datasets were merged prior to pre-processing with the MBEI algorithm, as in Subramanian and Simon [12]. Click here for file

Additional file 7 **Supplementary Figure S3**. Schematic overview of the methodology used to test sensitivity to differences in pre-processing in multi-gene biomarker performance. Click here for file

Additional file 8 **Supplementary Figure S4**. Results for all Cox proportional hazard ratio modeling analysis for the 24 different pre-processing schemes in the Director's Challenge dataset [21] for the six-gene biomarker are summarized in Forest plots. Boxes and lines are the hazard ratios and 95% confidence intervals, respectively. Patient classifications in all schemes are visualized in a heatmap. Rows represent pre-processing schedules, columns indicate patients. White indicates a patient predicted to have good prognosis, black indicates a patient predicted to have poor prognosis and gray indicates a patient that was not classified. Colored sidebar displays the different pre-processing schemes as explained in the legend. Click here for file

Additional file 9 **Supplementary Figure S5**. **(a) **Univariate analysis for each ProbeSet in the Director's Challenge dataset [21] revealed sensitivity to differences in pre-processing; the number of times a ProbeSet reached significance (*P*-value Wald test ≤ 0.05) in Cox proportional hazard ratio modeling analysis was highly variable. **(b, c) **Heatmaps of the hazard ratios (HRs) in each pre-processing schedule (columns) for ProbeSets (rows) with *P*-value Wald test ≤ 0.05 in at least one pre-processing schedule with default (b) or alternative annotation (c) also display this variance. Click here for file

Additional file 10 **Supplementary Figure S6**. **(a, b) **Marker performance improved when differentiating patients with identical classifications across all pre-processing schemes from the patients with ambiguous classifications for the three-gene biomarker (a versus b) in the Bild dataset [29]. Good prognosis patients are indicated by blue curves and poor prognosis patients by red curves in Kaplan-Meier plots. Hazard ratios and *P*-values are from stage-adjusted Cox proportional hazard ratio modeling followed by the Wald test. Click here for file
