# Supplementary material for: Exploiting the noise: improving biomarkers with ensembles of data analysis methodologies
Source: Genome Med. 2012 Nov 12;4(11):84. doi: 10.1186/gm385 (PMC3580418; doi:10.1186/gm385)
Supplement: Additional file 1 — Supplementary tables. Table S1: ProbeSet annotation used in analyses. Supplementary Table S2: overview of pre-processing algorithms. Supplementary Table S3: results of binary prediction performance of the three-gene and six-gene classifier in the Director's Challenge dataset. Supplementary Table S4: results (stage-adjusted) Cox proportional hazard ratio modeling three-gene classifier for all patients and stage IB patients in the 24 different pre-processed Director's Challenge datasets. Significant results (P < 0.05) are given in bold. Supplementary Table S5: results of binary prediction performance of the three-gene classifier in the 24 different pre-processed Director's Challenge datasets. Supplementary Table S6: results (stage-adjusted) Cox proportional hazard ratio modeling six-gene classifier for all patients and stage II patients in the 24 different pre-processed Director's Challenge datasets. Significant results (P < 0.05) are given in bold. Supplementary Table S7: results of binary prediction performance of the six-gene classifier in the 24 different pre-processed Director's Challenge datasets. Supplementary Table S8: results (stage-adjusted) Cox proportional hazard ratio modeling three-gene classifier for all patients in the 24 different pre-processed Bild datasets. Significant results (P < 0.05) are given in bold. Supplementary Table S9: results of binary prediction performance of the three-gene classifier in the 24 different pre-processed Bild datasets. Supplementary Table S10: results of binary prediction performance of the two classifiers in the Director's Challenge and Bild datasets for unanimous and ambiguous classified patients. [file gm385-S1.PDF]

Supplementary Table S1: ProbeSet annotation used in analyses

Gene Symbol	Entrez Gene ID	Affymetrix ProbeSet HGU133A	Affymetrix ProbeSet HGU133 PLUS2.0
**3-gene classifier**			
**CCR7**	1236	206337_at	206337_at
**HIF1A**	3091	200989_at	200989_at
**STX1A**	6804	204729_s_at	204729_s_at
**6-gene classifier**			
**Symbol**	**Entrez Gene ID**	**Affymetrix ProbeSet HGU133A**	
**CCT3**	7203	200910_at	
**HIF1A**	3091	200989_at	
**HLA-DPB1**	3115	201137_s_at	
**MAFK**	7975	206750_at	
**RNF5**	6048	209111_at	
**STX1A**	6804	204729_s_at	
**Housekeeping genes**			
**Symbol**	**Entrez Gene ID**	**Affymetrix ProbeSet HGU133A**	**Affymetrix ProbeSet HGU133 PLUS2.0**
**ACTB**	60	200801_x_at	200801_x_at<br>213867_x_at<br>224594_x_at
**B2M**	567	201891_s_at	201891_s_at<br>216231_s_at
**BAT1**	7919	200041_s_at	200041_s_at
**TBP**	6908	203135_at	203135_at

Supplementary Table S2: Overview of pre-processing algorithms

Algorithm	Background correction	Normalization	Summarization method
**RMA**	PM probe intensities are corrected array by array using a global model for the distribution of probe intensities.	Quantile normalization is used to impose the same empirical distribution of intensities to each array.	A multi-chip robust model (median polish) is fit to the log_2-transformed PM probes for a particular probeset to calculate expression values.
**GCRMA**	Probe sequences are used to calculate the background noise. PM probes are then corrected with MM probes.	Same as RMA.	Similar to RMA.
**MAS5**	Array is divided in K (16) grids. In each region the 2% lowest probe intensities are used to compute a background value. Each probe is adjusted based upon a weighted average of the backgrounds for each of the regions. Both PM and MM probes are corrected. The PM probes are corrected with MM probes.	Scaling: one array is used as baseline array; other arrays are scaled to have the same mean intensity.	Per array probe values for a particular probeset are combined with the Tukey-Biweight algorithm.
**MBEI**	No background correction is performed.	Invariantset normalization: one array is used as baseline array; arrays are normalized by selecting invariant sets of probes and using these to fit a non-linear relationship between baseline array and other arrays. The non-linear relation is then used to carry out the normalization.	A multi-chip model is fit to each probeset to calculate expression values.

PM probe = perfect match probe
MM probe = mismatch probe

Supplementary Table S3: results of binary prediction performance of the 3-gene and 6-gene classifier in the DC dataset.

Classifier	Patients	Sensitivity	Specificity	Accuracy	% unclassified
**3-gene**	**All**	0.51	0.56	0.54	-
**3-gene**	**Stage IA**	0.33	0.61	0.52	-
**3-gene**	**Stage IB**	0.65	0.53	0.58	-
**3-gene**	**Stage II**	0.60	0.61	0.60	-
**3-gene**	**Stage III**	0.61	0.56	0.60	-
**6-gene**	**All**	0.34	0.70	0.46	14
**6-gene**	**Stage IA**	0.19	0.76	0.50	12
**6-gene**	**Stage IB**	0.32	0.63	0.44	15
**6-gene**	**Stage II**	0.41	0.73	0.48	15
**6-gene**	**Stage III**	0.40	0.6	0.52	13

Supplementary Table S4: results (stage-adjusted) Cox proportional hazard ratio modeling 3-gene classifier for all patients and stage IB patients in the 24 different pre-processed DC datasets. Significant results ($p < 0.05$) are given in bold.

Pre-processing				All patients		Stage IB patients	
Algorithm	Dataset	Annotation	Transform	HR [95% CI]	P	HR [95% CI]	P
RMA	Merged	Alternative	log_2	**1.42 [1.06 - 1.92]**	**2.07×10^{-2}**	1.62 [0.93 - 2.82]	8.87×10^{-2}
RMA	Merged	Default	log_2	**1.64 [1.22 - 2.21]**	**1.11×10^{-3}**	**1.80 [1.03 - 3.14]**	**3.97×10^{-2}**
RMA	Separate	Alternative	log_2	1.23 [0.92 - 1.64]	1.72×10^{-1}	1.44 [0.83 - 2.49]	1.90×10^{-1}
RMA	Separate	Default	log_2	**1.63 [1.21 - 2.19]**	**1.18×10^{-3}**	**2.05 [1.16 - 3.62]**	**1.41×10^{-2}**
GCRMA	Merged	Alternative	log_2	**1.38 [1.03 - 1.85]**	**2.88×10^{-2}**	1.29 [0.75 - 2.23]	3.55×10^{-1}
GCRMA	Merged	Default	log_2	**1.40 [1.05 - 1.88]**	**2.38×10^{-2}**	1.54 [0.89 - 2.66]	1.20×10^{-1}
GCRMA	Separate	Alternative	log_2	**1.39 [1.04 - 1.87]**	**2.76×10^{-2}**	1.21 [0.70 - 2.09]	5.01×10^{-1}
GCRMA	Separate	Default	log_2	**1.43 [1.06 - 1.91]**	**1.78×10^{-2}**	1.54 [0.90 - 2.66]	1.18×10^{-1}
MAS5	Merged	Alternative	log_2	**1.41 [1.05 - 1.90]**	**2.33×10^{-2}**	**1.90 [1.06 - 3.39]**	**3.01×10^{-2}**
MAS5	Merged	Default	log_2	**1.46 [1.08 - 1.97]**	**1.42×10^{-2}**	**1.84 [1.04 - 3.26]**	**3.65×10^{-2}**
MAS5	Separate	Alternative	log_2	1.34 [0.99 - 1.80]	5.18×10^{-2}	1.37 [0.78 - 2.40]	2.69×10^{-1}
MAS5	Separate	Default	log_2	**1.55 [1.15 - 2.08]**	**4.10×10^{-3}**	**1.82 [1.03 - 3.19]**	**3.85×10^{-2}**
MAS5	Merged	Alternative	None	**1.57 [1.17 - 2.12]**	**3.07×10^{-3}**	**1.93 [1.10 - 3.39]**	**2.27×10^{-2}**
MAS5	Merged	Default	None	**1.85 [1.37 - 2.50]**	**6.01×10^{-5}**	**1.79 [1.02 - 3.12]**	**4.20×10^{-2}**
MAS5	Separate	Alternative	None	**1.57 [1.17 - 2.11]**	**3.02×10^{-3}**	1.59 [0.91 - 2.77]	1.01×10^{-1}
MAS5	Separate	Default	None	**1.49 [1.11 - 1.99]**	**7.79×10^{-3}**	1.68 [0.97 - 2.92]	6.66×10^{-2}
MBEI	Merged	Alternative	log_2	1.15 [0.86 - 1.54]	3.48×10^{-1}	1.19 [0.69 - 2.06]	5.37×10^{-1}
MBEI	Merged	Default	log_2	1.28 [0.96 - 1.72]	9.70×10^{-2}	1.14 [0.66 - 1.97]	6.45×10^{-1}
MBEI	Separate	Alternative	log_2	1.23 [0.92 - 1.65]	1.69×10^{-1}	1.23 [0.71 - 2.13]	4.62×10^{-1}
MBEI	Separate	Default	log_2	**1.36 [1.02 - 1.83]**	**3.88×10^{-2}**	1.32 [0.76 - 2.29]	3.23×10^{-1}
MBEI	Merged	Alternative	None	1.32 [0.98 - 1.76]	6.58×10^{-2}	1.23 [0.71 - 2.11]	4.66×10^{-1}
MBEI	Merged	Default	None	**1.34 [1.00 - 1.80]**	**4.74×10^{-2}**	1.10 [0.64 - 1.90]	7.25×10^{-1}
MBEI	Separate	Alternative	None	1.22 [0.91 - 1.63]	1.82×10^{-1}	0.89 [0.52 - 1.53]	6.72×10^{-1}
MBEI	Separate	Default	None	1.28 [0.95 - 1.72]	1.02×10^{-1}	0.94 [0.54 - 1.62]	8.20×10^{-1}

Supplementary Table S5: results of binary prediction performance of the 3-gene classifier in the 24 different pre-processed DC datasets.

Pre-processing						
Algorithm	Dataset	Annotation	Transform	Sensitivity	Specificity	Accuracy
RMA	Merged	Alternative	log_2	0.60	0.56	0.58
RMA	Merged	Default	log_2	0.60	0.57	0.59
RMA	Separate	Alternative	log_2	0.51	0.56	0.54
RMA	Separate	Default	log_2	0.58	0.57	0.58
GCRMA	Merged	Alternative	log_2	0.49	0.63	0.56
GCRMA	Merged	Default	log_2	0.50	0.61	0.56
GCRMA	Separate	Alternative	log_2	0.47	0.66	0.56
GCRMA	Separate	Default	log_2	0.47	0.67	0.56
MAS5	Merged	Alternative	log_2	0.60	0.54	0.57
MAS5	Merged	Default	log_2	0.61	0.53	0.57
MAS5	Separate	Alternative	log_2	0.57	0.53	0.55
MAS5	Separate	Default	log_2	0.59	0.57	0.58
MAS5	Merged	Alternative	None	0.59	0.60	0.59
MAS5	Merged	Default	None	0.60	0.60	0.60
MAS5	Separate	Alternative	None	0.55	0.62	0.58
MAS5	Separate	Default	None	0.54	0.59	0.56
MBEI	Merged	Alternative	log_2	0.54	0.51	0.53
MBEI	Merged	Default	log_2	0.57	0.47	0.52
MBEI	Separate	Alternative	log_2	0.53	0.54	0.54
MBEI	Separate	Default	log_2	0.56	0.54	0.55
MBEI	Merged	Alternative	None	0.54	0.56	0.55
MBEI	Merged	Default	None	0.55	0.52	0.54
MBEI	Separate	Alternative	None	0.50	0.55	0.52
MBEI	Separate	Default	None	0.50	0.58	0.54

Supplementary Table S6: results (stage-adjusted) Cox proportional hazard ratio modeling 6-gene classifier for all patients and stage II patients in the 24 different pre-processed DC datasets. Significant results (p < 0.05) are given in bold.

Pre-processing				All patients		Stage II patients		
Algorithm	Dataset	Annotation	Transform	HR [95% CI]	P	HR [95% CI]	P	% unclassified
RMA	Merged	Alternative	log_2	**1.49 [1.08 - 2.04]**	**1.36×10^{-2}**	**2.55 [1.40 - 4.66]**	**2.34×10^{-3}**	13
RMA	Merged	Default	log_2	**1.42 [1.03 - 1.97]**	**3.13×10^{-2}**	**1.95 [1.05 - 3.63]**	**3.53×10^{-2}**	16
RMA	Separate	Alternative	log_2	**1.43 [1.04 - 1.95]**	**2.70×10^{-2}**	**2.18 [1.21 - 3.91]**	**9.28×10^{-3}**	9
RMA	Separate	Default	log_2	**1.42 [1.04 - 1.96]**	**3.01×10^{-2}**	**1.90 [1.05 - 3.44]**	**3.50×10^{-2}**	14
GCRMA	Merged	Alternative	log_2	**1.44 [1.06 - 1.96]**	**2.13×10^{-2}**	1.83 [0.99 - 3.39]	5.27×10^{-2}	10
GCRMA	Merged	Default	log_2	1.33 [0.97 - 1.83]	7.56×10^{-2}	1.39 [0.75 - 2.56]	2.91×10^{-1}	15
GCRMA	Separate	Alternative	log_2	1.32 [0.96 - 1.81]	8.74×10^{-2}	1.41 [1.00 - 3.15]	5.18×10^{-2}	11
GCRMA	Separate	Default	log_2	1.18 [0.86 - 1.63]	3.10×10^{-1}	1.41 [0.78 - 2.54]	2.52×10^{-1}	14
MAS5	Merged	Alternative	log_2	**1.44 [1.03 - 2.01]**	**3.29×10^{-2}**	**2.06 [1.10 - 3.87]**	**2.26×10^{-2}**	22
MAS5	Merged	Default	log_2	1.56 [1.13 - 2.17]	7.09×10^{-3}	1.73 [0.96 - 3.15]	7.04×10^{-2}	26
MAS5	Separate	Alternative	log_2	1.33 [0.96 - 1.85]	9.00×10^{-2}	**2.03 [1.12 - 3.66]**	**1.93×10^{-2}**	22
MAS5	Separate	Default	log_2	1.37 [0.99 - 1.90]	5.98×10^{-2}	**1.90 [1.06 - 3.39]**	**3.10×10^{-2}**	21
MAS5	Merged	Alternative	None	**2.77 [1.62 - 4.76]**	**2.12×10^{-4}**	**5.59 [2.50 - 12.53]**	**2.86×10^{-5}**	21
MAS5	Merged	Default	None	**2.55 [1.50 - 4.33]**	**5.17×10^{-4}**	**5.28 [2.48 - 11.23]**	**1.55×10^{-5}**	23
MAS5	Separate	Alternative	None	**2.36 [1.35 - 4.11]**	**2.45×10^{-3}**	**4.57 [2.05 - 10.20]**	**2.01×10^{-4}**	18
MAS5	Separate	Default	None	**1.88 [1.06 - 3.33]**	**3.19×10^{-2}**	**5.58 [2.39 - 13.03]**	**7.05×10^{-5}**	20
MBEI	Merged	Alternative	log_2	1.12 [0.82 - 1.54]	4.61×10^{-1}	1.44 [0.79 - 2.65]	2.38×10^{-1}	16
MBEI	Merged	Default	log_2	1.00 [0.72 - 1.40]	9.85×10^{-1}	1.57 [0.83 - 2.97]	1.66×10^{-1}	16
MBEI	Separate	Alternative	log_2	1.21 [0.89 - 1.64]	2.33×10^{-1}	1.59 [0.89 - 2.84]	1.19×10^{-1}	16
MBEI	Separate	Default	log_2	1.13 [1.82 - 1.55]	4.50×10^{-1}	1.79 [0.98 - 3.25]	5.72×10^{-2}	16
MBEI	Merged	Alternative	None	**2.18 [1.27 - 3.72]**	**4.50×10^{-3}**	**2.39 [1.01 - 5.66]**	**4.83×10^{-2}**	9
MBEI	Merged	Default	None	**2.47 [1.14 - 5.34]**	**2.23×10^{-2}**	1.73 [0.22 - 13.57]	6.04×10^{-1}	11
MBEI	Separate	Alternative	None	**1.71 [1.02 - 2.87]**	**4.37×10^{-2}**	1.89 [0.80 - 4.46]	1.47×10^{-1}	9
MBEI	Separate	Default	None	**2.17 [1.17 - 4.03]**	**1.43×10^{-2}**	**3.55 [1.38 - 9.15]**	**8.77×10^{-3}**	9

Supplementary Table S7: results of binary prediction performance of the 6-gene classifier in the 24 different pre-processed DC datasets.

Pre-processing							
Algorithm	Dataset	Annotation	Transform	Sensitivity	Specificity	Accuracy	% unclassified
RMA	Merged	Alternative	log_2	0.33	0.72	0.47	13
RMA	Merged	Default	log_2	0.34	0.70	0.47	16
RMA	Separate	Alternative	log_2	0.35	0.70	0.47	9
RMA	Separate	Default	log_2	0.34	0.70	0.46	14
GCRMA	Merged	Alternative	log_2	0.43	0.66	0.51	10
GCRMA	Merged	Default	log_2	0.43	0.63	0.50	15
GCRMA	Separate	Alternative	log_2	0.42	0.67	0.50	11
GCRMA	Separate	Default	log_2	0.41	0.64	0.49	14
MAS5	Merged	Alternative	log_2	0.32	0.81	0.48	22
MAS5	Merged	Default	log_2	0.35	0.81	0.51	26
MAS5	Separate	Alternative	log_2	0.33	0.76	0.47	22
MAS5	Separate	Default	log_2	0.32	0.74	0.47	21
MAS5	Merged	Alternative	None	0.25	0.88	0.46	21
MAS5	Merged	Default	None	0.28	0.84	0.47	23
MAS5	Separate	Alternative	None	0.21	0.84	0.44	18
MAS5	Separate	Default	None	0.21	0.82	0.44	20
MBEI	Merged	Alternative	log_2	0.26	0.75	0.42	16
MBEI	Merged	Default	log_2	0.25	0.73	0.41	16
MBEI	Separate	Alternative	log_2	0.27	0.72	0.42	16
MBEI	Separate	Default	log_2	0.28	0.74	0.42	16
MBEI	Merged	Alternative	None	0.14	0.84	0.38	9
MBEI	Merged	Default	None	0.11	0.90	0.38	11
MBEI	Separate	Alternative	None	0.14	0.84	0.38	9
MBEI	Separate	Default	None	0.12	0.83	0.37	9

Supplementary Table S8: results (stage-adjusted) Cox proportional hazard ratio modeling 3-gene classifier for all patients in the 24 different pre-processed Bild datasets. Significant results ($p < 0.05$) are given in bold.

Pre-processing				All patients	
Algorithm	Dataset	Annotation	Transform	HR [95% CI]	P
RMA	Merged	Alternative	log_2	1.54 [0.83 - 2.86]	1.69×10^{-1}
RMA	Merged	Default	log_2	1.02 [0.58 - 1.79]	9.43×10^{-1}
RMA	Separate	Alternative	log_2	1.08 [0.60 - 1.94]	7.97×10^{-1}
RMA	Separate	Default	log_2	1.23 [0.71 - 2.13]	4.66×10^{-1}
GCRMA	Merged	Alternative	log_2	1.06 [0.58 - 1.94]	8.43×10^{-1}
GCRMA	Merged	Default	log_2	0.99 [0.55 - 1.77]	9.68×10^{-1}
GCRMA	Separate	Alternative	log_2	0.94 [0.51 - 1.73]	8.44×10^{-1}
GCRMA	Separate	Default	log_2	0.99 [0.55 - 1.77]	9.74×10^{-1}
MAS5	Merged	Alternative	log_2	0.96 [0.52 - 1.76]	8.87×10^{-1}
MAS5	Merged	Default	log_2	1.00 [0.57 - 1.76]	9.89×10^{-1}
MAS5	Separate	Alternative	log_2	1.10 [0.64 - 1.90]	7.25×10^{-1}
MAS5	Separate	Default	log_2	1.09 [0.63 - 1.90]	7.56×10^{-1}
MAS5	Merged	Alternative	None	1.64 [0.94 - 2.86]	8.48×10^{-2}
MAS5	Merged	Default	None	1.65 [0.90 - 3.03]	1.03×10^{-1}
MAS5	Separate	Alternative	None	1.54 [0.89 - 2.69]	1.25×10^{-1}
MAS5	Separate	Default	None	1.36 [0.77 -2.40]	2.95×10^{-1}
MBEI	Merged	Alternative	log_2	1.10 [0.60 - 2.00]	7.59×10^{-1}
MBEI	Merged	Default	log_2	1.05 [0.57 - 1.94]	8.76×10^{-1}
MBEI	Separate	Alternative	log_2	1.07 [0.60 - 1.92]	8.16×10^{-1}
MBEI	Separate	Default	log_2	0.85 [0.46 - 1.57]	6.06×10^{-1}
MBEI	Merged	Alternative	None	1.13 [0.62 - 2.04]	6.91×10^{-1}
MBEI	Merged	Default	None	**2.15 [1.15 - 4.03]**	$\mathbf{1.67 \times 10^{-2}}$
MBEI	Separate	Alternative	None	1.02 [0.57 - 1.84]	9.44×10^{-1}
MBEI	Separate	Default	None	1.09 [0.61 - 1.95]	7.79×10^{-1}

Supplementary Table S9: results of binary prediction performance of the 3-gene classifier in the 24 different pre-processed Bild datasets.

Pre-processing						
Algorithm	Dataset	Annotation	Transform	Sensitivity	Specificity	Accuracy
RMA	Merged	Alternative	$\log_2$	0.60	0.47	0.58
RMA	Merged	Default	$\log_2$	0.57	0.40	0.53
RMA	Separate	Alternative	$\log_2$	0.57	0.40	0.53
RMA	Separate	Default	$\log_2$	0.57	0.60	0.58
GCRMA	Merged	Alternative	$\log_2$	0.52	0.47	0.51
GCRMA	Merged	Default	$\log_2$	0.48	0.53	0.49
GCRMA	Separate	Alternative	$\log_2$	0.50	0.47	0.49
GCRMA	Separate	Default	$\log_2$	0.48	0.53	0.49
MAS5	Merged	Alternative	$\log_2$	0.57	0.47	0.55
MAS5	Merged	Default	$\log_2$	0.53	0.53	0.53
MAS5	Separate	Alternative	$\log_2$	0.52	0.53	0.52
MAS5	Separate	Default	$\log_2$	0.53	0.53	0.53
MAS5	Merged	Alternative	None	0.53	0.53	0.53
MAS5	Merged	Default	None	0.50	0.53	0.51
MAS5	Separate	Alternative	None	0.50	0.60	0.52
MAS5	Separate	Default	None	0.50	0.47	0.49
MBEI	Merged	Alternative	$\log_2$	0.66	0.33	0.59
MBEI	Merged	Default	$\log_2$	0.60	0.33	0.55
MBEI	Separate	Alternative	$\log_2$	0.60	0.33	0.55
MBEI	Separate	Default	$\log_2$	0.59	0.27	0.52
MBEI	Merged	Alternative	None	0.62	0.40	0.58
MBEI	Merged	Default	None	0.66	0.47	0.62
MBEI	Separate	Alternative	None	0.53	0.40	0.51
MBEI	Separate	Default	None	0.55	0.27	0.49

Supplementary Table S10: results of binary prediction performance of the 3-gene and 6-gene classifier for unanimous and ambiguous patient classifications.

Dataset	Patient sub group		Sensitivity	Specificity	Accuracy	# patients
DC	Unanimous classification	3-gene	0.64	0.60	0.63	122
DC	Ambiguous classification	3-gene	0.54	0.56	0.55	236
DC	Unanimous classification	6-gene	0.12	0.98	0.42	115
DC	Ambiguous classification	6-gene	0.47	0.52	0.48	194*
Bild	Unanimous classification	3-gene	0.68	0.50	0.64	28
Bild	Ambiguous classification	3-gene	0.50	0.67	0.53	45

* 56 patients unclassified were disregarded
