# Supplementary figures and images for: Exploiting the noise: improving biomarkers with ensembles of data analysis methodologies
Source: Genome Med. 2012 Nov 12;4(11):84. doi: 10.1186/gm385 (PMC3580418; doi:10.1186/gm385)

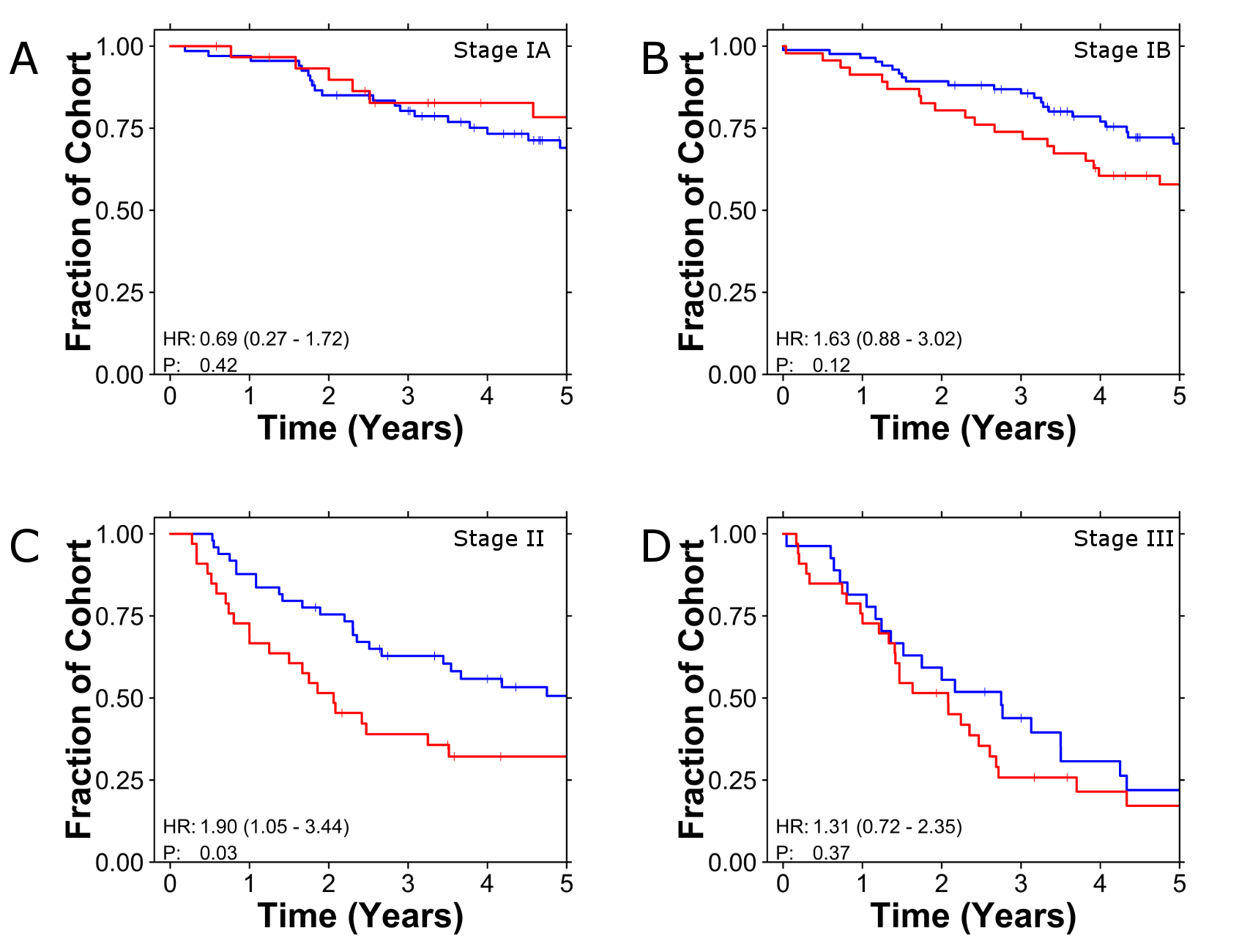

Supplement: Additional file 5 — Supplementary Figure S1. (a-d) Performance of the six-gene biomarker was evaluated in a sub-stage analysis (stage IA (a), stage IB (b), stage II (c), and stage III (d) patients), which were visualized with Kaplan-Meier curves. Each patient was classified into good (blue curves) and poor (red curves) prognosis groups using the six-gene biomarker. Hazard ratios and P-values are from Cox proportional hazard ratio modeling followed by the Wald test. [file gm385-S5.TIFF]

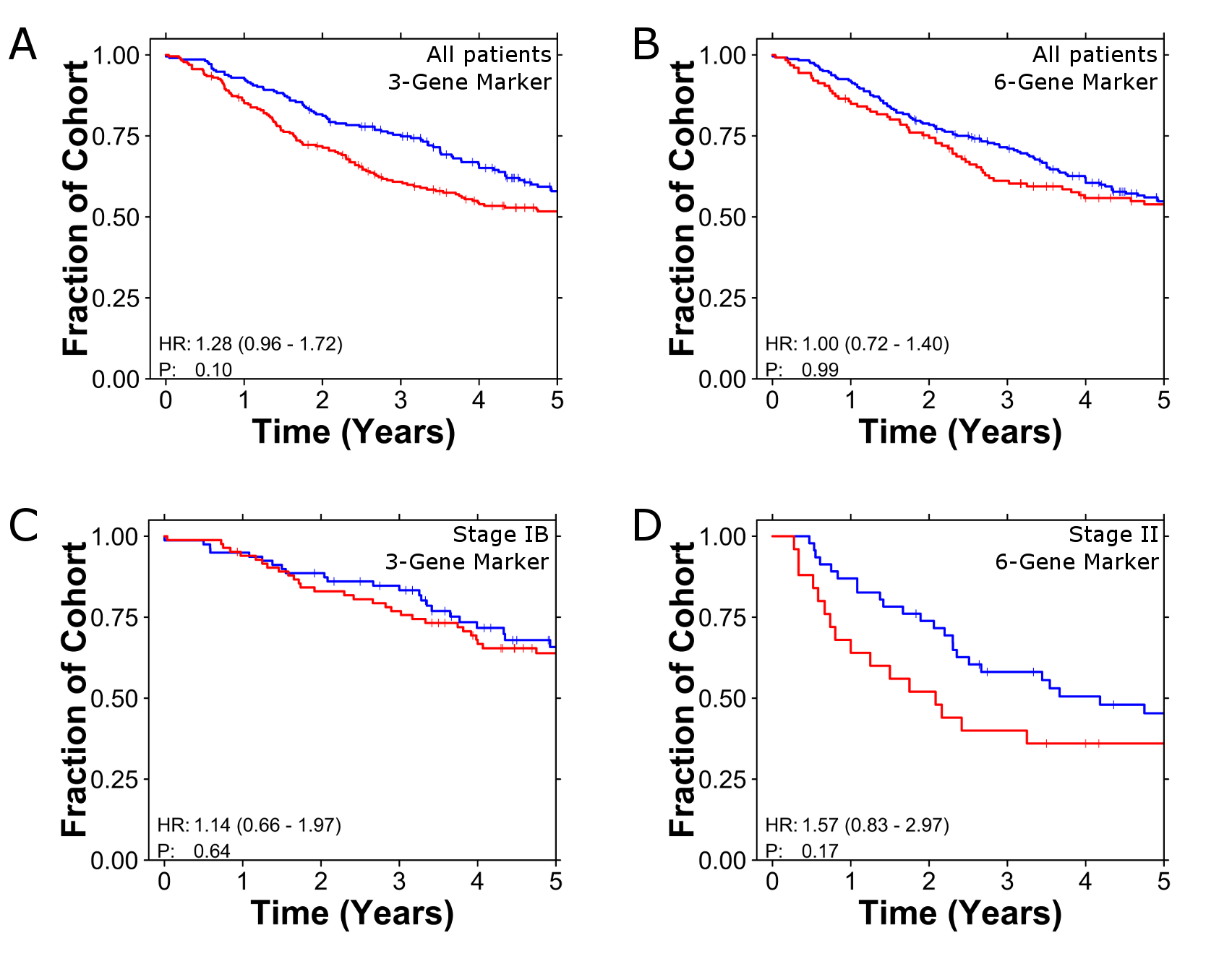

Supplement: Additional file 6 — Supplementary Figure S2. (a-d) Kaplan-Meier curves for the three-gene (a: all patients; c: stage IB) and six-gene (b: all patients; d: stage II) classifiers in the Director's Challenge data [21], where datasets were merged prior to pre-processing with the MBEI algorithm, as in Subramanian and Simon [12]. [file gm385-S6.TIFF]

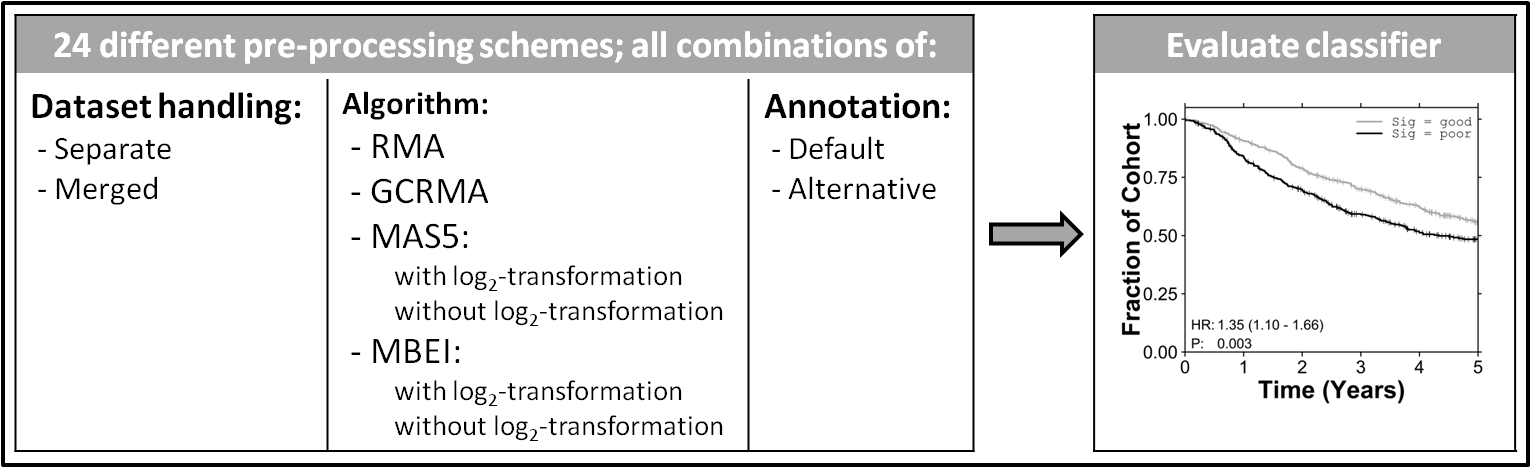

Supplement: Additional file 7 — Supplementary Figure S3. Schematic overview of the methodology used to test sensitivity to differences in pre-processing in multi-gene biomarker performance. [file gm385-S7.TIFF]

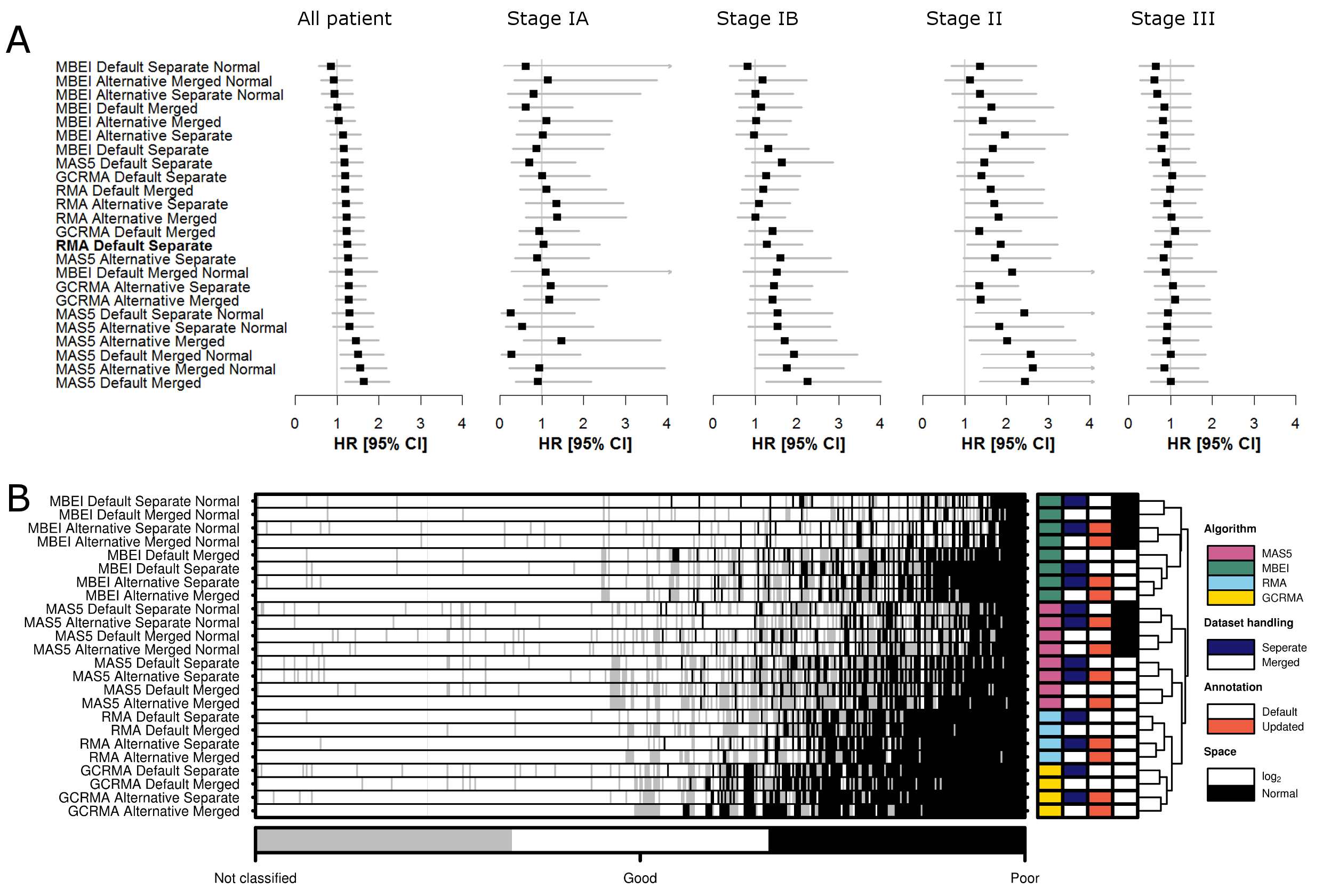

Supplement: Additional file 8 — Supplementary Figure S4. Results for all Cox proportional hazard ratio modeling analysis for the 24 different pre-processing schemes in the Director's Challenge dataset [21] for the six-gene biomarker are summarized in Forest plots. Boxes and lines are the hazard ratios and 95% confidence intervals, respectively. Patient classifications in all schemes are visualized in a heatmap. Rows represent pre-processing schedules, columns indicate patients. White indicates a patient predicted to have good prognosis, black indicates a patient predicted to have poor prognosis and gray indicates a patient that was not classified. Colored sidebar displays the different pre-processing schemes as explained in the legend. [file gm385-S8.TIFF]

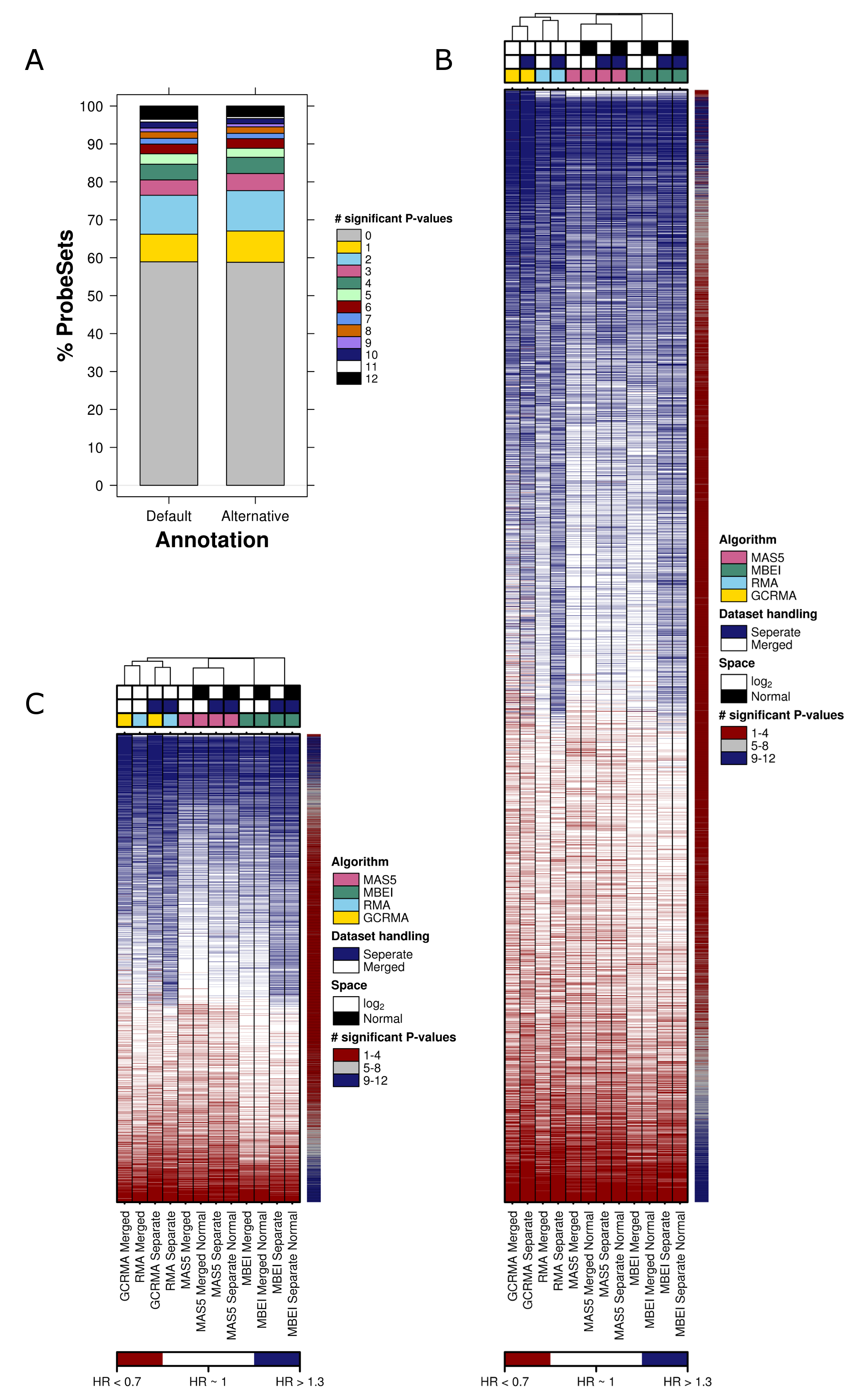

Supplement: Additional file 9 — Supplementary Figure S5. (a) Univariate analysis for each ProbeSet in the Director's Challenge dataset [21] revealed sensitivity to differences in pre-processing; the number of times a ProbeSet reached significance (P-value Wald test ≤ 0.05) in Cox proportional hazard ratio modeling analysis was highly variable. (b, c) Heatmaps of the hazard ratios (HRs) in each pre-processing schedule (columns) for ProbeSets (rows) with P-value Wald test ≤ 0.05 in at least one pre-processing schedule with default (b) or alternative annotation (c) also display this variance. [file gm385-S9.TIFF]

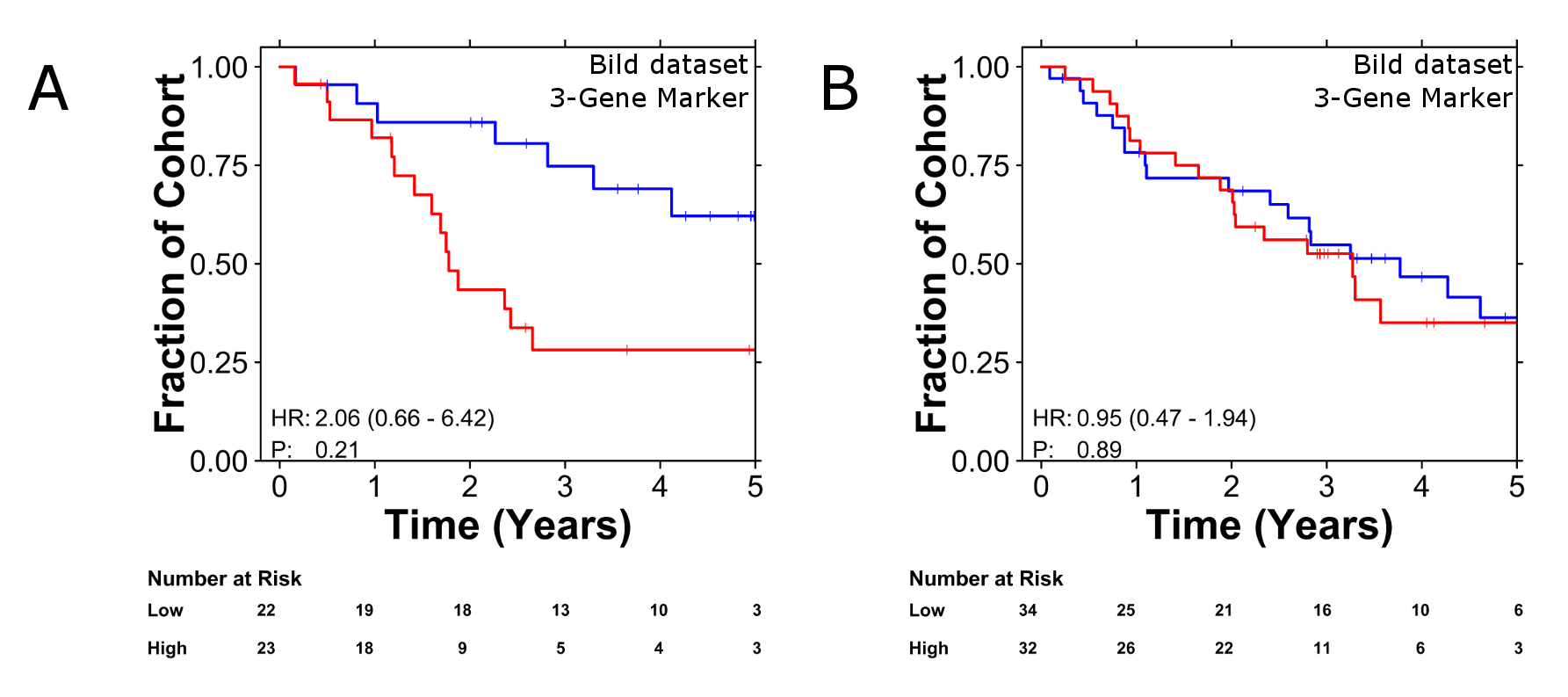

Supplement: Additional file 10 — Supplementary Figure S6. (a, b) Marker performance improved when differentiating patients with identical classifications across all pre-processing schemes from the patients with ambiguous classifications for the three-gene biomarker (a versus b) in the Bild dataset [29]. Good prognosis patients are indicated by blue curves and poor prognosis patients by red curves in Kaplan-Meier plots. Hazard ratios and P-values are from stage-adjusted Cox proportional hazard ratio modeling followed by the Wald test. [file gm385-S10.TIFF]
